# Carrot and Stick Approach: The Exploitative Leadership and Absenteeism in Education Sector

**DOI:** 10.3389/fpsyg.2022.890064

**Published:** 2022-07-20

**Authors:** Muhammad Waheed Akhtar, Chunhui Huo, Fauzia Syed, Muhammad Arslan Safdar, Arsalan Rasool, Mudassir Husnain, Muhammad Awais, Muhammad Shoaib Sajjad

**Affiliations:** ^1^Asia-Australia Business College, Liaoning University, Shenyang, China; ^2^Department of Management Sciences, COMSATS University Islamabad, Sahiwal, Pakistan; ^3^Faculty of Management Sciences, International Islamic University, Islamabad, Pakistan; ^4^Department of Public Administration, The Islamia University Bahawalpur, Bahawalpur, Pakistan; ^5^UE Business School, University of Education, Lahore, Pakistan; ^6^Faculty of Management Sciences, The University of Lahore, Sargodha, Pakistan

**Keywords:** exploitative leadership, facades of conformity, depression, absenteeism, education sector

## Abstract

Utilizing the conservation of resources theory, this study investigates serial mediation of facades of conformity and depression between exploitative leadership and absenteeism. A total of 211 education sector employees using the convenient sampling technique took part in the survey with data collected in a time-lagged research design. Findings of the study reveal that facades of conformity and depression mediate the independent paths and play a serial mediating role between EL and absenteeism path. This study suggests that EL works as a workplace stressor, under which employees try to protect their valuable resources from further loss in the form of facades of conformity, in doing so, it leads to depression; thus, employees ultimately use absenteeism as an active coping strategy to cope with workplace stressors.

## Introduction

Leadership plays an important role in shaping employees’ behavior at the workplace and is vital for organizational success (Yukl, 2012). Recently, organizational researchers have started focusing on the negative side of leadership (Schyns and Schilling, 2013) especially in education sector (Akhtar et al., 2021b; De Clercq et al., 2021, 2022). This negative or dark side of leadership is marred with emerging new constructs and multiple labels such as abusive supervision (Tepper, 2000), petty tyranny (Ashforth, 1994), despotic leadership (De Hoogh and Den Hartog, 2008; Syed et al., 2020), destructive leadership (Krasikova et al., 2013; Schyns and Schilling, 2013), and exploitative leadership (EL) (Schmid et al., 2019b). Akhtar et al. (2021b) investigated the effect of dark leadership on employee outcomes with mediation and moderation models in education sector.

Exploitative leadership mostly encompasses the features of destructive leadership (Schmid et al., 2018). EL is defined as “*leadership with the primary intention to further the leader’s self-interest by exploiting others, reflected in five dimensions: genuine egoistic behaviors, taking credit, exerting pressure, undermining development, and manipulating*” (Schmid et al., 2019b). Recently, Schmid et al. (2019b) debated on the concept of EL as a prevalent negative leadership behavior targeting the followers; however, it is void of inherent hostility or aggressiveness. EL is different from other forms of negative leadership due to its distinctive factors. First, the exploitative leader is usually high in self-interest, and she/he is more likely to act egoistically. She/he mostly prioritizes her/his goals over subordinates’ needs and takes the credit of subordinate’s works (Schmid et al., 2019b). Second, exploitative leaders pressurize their subordinates by using influential tactics or manipulating them such as overt aggression and create rivalry among subordinates to benefit herself/himself (Schmid et al., 2019b). Third, exploitative leaders behave friendly and delegate additional tasks among subordinates, even if they are already burdened and overloaded (Schmid et al., 2019b). Fourth, exploitative leaders underchallenge the subordinates by delegating the tedious tasks among them and hinder their career advancements (Schmid et al., 2019b).

Exploitative leadership integrates with a broad range of dysfunctional outcomes observed at the individual level. There has been consistent efforts invested to examine mediated models, as by doing so, studies are able to directly examine theoretical mechanisms, i.e., how employees get avenged due to dark supervision (Tepper et al., 2017). Schmid et al. (2019a) investigated exploitative leaders’ impact on the different individual levels (e.g., job satisfaction, commitment, burnout, and workplace deviance behavior). Schmid et al. (2018) apprised in their study that how EL plays an adversal role on employee’s emotional reactions (i.e., negative affect) and turnover intentions. Syed et al. (2021) stated that EL is a stressor that dampens the employee job performance and creativity through knowledge hiding. Despite this plethora of research, we know very less about how EL works and converts into different employee outcomes such as knowledge hiding, psychological distress, and turnover intentions. Thus, very less attention has been devoted to uncover the consequences of EL, especially how EL may influence employee absenteeism, “*as a form of withdrawal behavior whereby employees avoid unfavorable work situations by not showing up for work*” (Harrison and Martocchio, 1998). As employee absenteeism is very dangerous for organization (Bowen, 1982), by practicing it employees try to enhance a distance between the organization and themselves (Farrell and Petersen, 1984). Therefore, the intent of this research was to explore the effect of EL on employee absenteeism from the lens of conservation of resources (COR) theory. As Hobfoll (1989) stated, normally people attempt to protect their valuable resources from further loss when encountered threatening situations. EL is a workplace stressor. Hence, when a leader behaves exploitatively, then the follower engage in absenteeism in order to protect their valued resources. In addition to the direct effect of EL to absenteeism, we proposed two mediating mechanisms, namely, facades of conformity (FOC), “*false representations created by organization members to appear as if they embrace organizational values*” (Hewlin, 2003), and depression, “*as a common mental health issue in which the individual feels fatigued as well as sad and loses interest in everything*” Kroenke et al. (2001), under COR assumptions. According to the study by Hobfoll (1989), individuals try to gain and protect their valuable resources. The underlying principle of the theory of COR is “*individuals strive to retain, protect, and foster those things that they value*” (Hobfoll, 2001; Westman et al., 2004). Hobfoll corroborates that employees are susceptible and likely protect their numerous valued resources ranging from object resources to energy and from personal to condition based (Hobfoll, 1989, 2001). Exploitative leaders are usually the source of pressure and a threat to resources loss due to their self-interested characteristics and tendencies (e.g., egoistic, manipulative, taking credit, exerting pressure, and undermining the followers), which leads workers to hide their emotions and mask themselves (FOC) (Hobfoll, 1989, 2001, 2011). The exploitative leaders exert pressure and undermine against the personal favors he/she provided to their followers; therefore, followers may indulge in conformity to avoid punishment (Aycan, 2006). In the end, hiding an internal resource (i.e., adoption of facades) may cause psychological suffering such as depression among followers, and they may detach (i.e., absenteeism) themselves from their work (Hobfoll, 2011). Followers become frustrated in a situation where exploitative leaders demand undue favors and loyalty (Soylu, 2011), which deteriorate of their resources; therefore, they engage in absenteeism.

Through our application of COR theory as an overarch to uncover the EL-absenteeism relationship, we sought several contributions in the literature. First, recent studies have examined the EL and employee behaviors (Schmid et al., 2018, 2019a); we extended the literature on EL by positing that the FOC may act as the underlying mechanism by which subordinates protect their valuable resources from loss and engage in absenteeism due to EL. We drew upon COR theory (Hobfoll, 1989) and opined that employees “*strive to retain, protect, and build resources and that what is threatening to them is the potential or actual loss of these valued resources*” (p. 516). Hobfoll defined resources as “*those objects, personal characteristics, conditions, or energies that are valued in their own right, or that are valued because they act as conduits to the achievement or protection of valued resources*” (2001). Strains occur if people experience a threat to one of their resources, an actual resource loss, or a lack of resource gain after a resource investment. Then, exploited employees might engage in increased FOC (i.e., masking themselves) and opt absenteeism behavior, just to protect their resources from exploitation.

Second, this study contributes by assessing how depression mediates the relationship of EL and employee absenteeism; subsequently, FOC and depression serially mediate the relationship between EL and employee absenteeism. In situations where the workers perceive that there is a contradiction in their values with that of their organization, they mostly pretend that they are fit into the organization (Hewlin et al., 2017). Due to work pressures or work stressor employees, publicly, they may indulge in such types of behavior which are not real or original (masking themselves) (Hewlin, 2003). This further leads to the hampering of the employee mental health/cognition in the form of depression and finally leads to employees’ absenteeism. The employee use it as a coping strategy just to protect their valuable resources from the exploitative boss. In particular, we focused on EL and absenteeism’s relationship that is serially mediated by FOC and depression. Finally, this inquiry is built on a lagged design research design, which is likely to minimize the threat of common method bias (CMB) (Podsakoff et al., 2003).

## Theory and Hypotheses

### Exploitative Leadership and Absenteeism

Exploitative leaders being high on self-interest build all the connections with their subordinates based on personal gains and interests; moreover, such leaders tend to utilize their followers to achieve self-centered objectives (Schmid et al., 2019b). Schilling (2009) stated that exploitative leaders usually adopt a carrot and stick approach, i.e., fear strategy, and exploit their followers through extrinsic rewards to achieve personal and organizational goals. Voluntary absence from the workplace occurring due to domestic pressures and sudden ailing of the employees might yield harmful ramification in employees such as workplace stress, diminished self-confidence/self-esteem, and workplace maltreatment (Lach, 1999). Such employees, when faced exploitative leaders at the workplace, indulged into unfavorable outcomes, including high turnover intentions and low organizational commitment (Schmid et al., 2018, 2019b; Syed et al., 2019a), job performance, and creativity (Syed et al., 2019a).

Tepper et al. (2006) reported that dark leadership yields negative consequences in employees, such as low productivity, high absenteeism, and hospitalization costs. Exploitative leaders being focused on self-interest behave egoistically, take undue credit, exert pressure, and manipulate the followers. These action tendencies may make the workplace stressful, and followers adopt absenteeism as a coping strategy to protect their valuable resources from exploitative leaders. Previous research also corroborates that to cope with stressful work environments, employees usually take short breaks such as temporary or short-term absenteeism from the work settings (Hassan et al., 2014).

Based on COR theory (Hobfoll, 1989), this study proposes that EL works as a workplace stressor; employees initially engage in efforts to meet an exploitative leader’s undue demands. Employees’ behavioral reactions to poor work conditions such EL, as a way of mitigating the resource loss caused by EL, increase when relevant personal qualities increase the desirability of preventing more resource drainage, according to the COR mechanisms (Hobfoll, 2001). It becomes very taxing for subordinates of such exploitative leaders, and they are unable to recover and replenish their resource pool, which results in energy depletion. Thus, such employees engage in absenteeism as a coping strategy to save their energy and continue their work. Hence, it is proposed that:

H1: *Exploitative leadership (EL) is positively related to employee absenteeism.*

### Facade of Conformity as a Mediator

Hewlin (2009) in their study stated that perceived non-participative work environments, minority status, self-monitoring, and collectivism were significantly related to creating FOC. Although some antecedents of facade creation have been discussed in the literature, e.g., subjectivity in the organizational reward system and leader’s integrity (Hewlin et al., 2017), we do not know much about why and how it operates. The dark leadership types have been deleteriously associated with a range of employee perceptions, behaviors, and workplace outcomes (Mackey et al., 2017). Creating facades is also a result of diminished self-esteem among organizational members (Mitchell et al., 2015). So exploitative leaders discourage his/her followers by acting egoistically, manipulating, exerting pressure on them, and taking credit for their efforts, that is why followers will indulge in the process of FOC.

Creating FOC, in actuality, is the reflection that the employees are prone to the environment, which leads them to suppress their feelings and view to cope with the stress and show their submissiveness to the organizational values beliefs (Hewlin et al., 2016). The result of this blitz is that it can end up in the individual’s retaliation (i.e., direct or indirect) against the organization. It continuously presses him/her to engage in facade creation, which contradicts his/her true values and core beliefs (Hewlin et al., 2016).

Exploitative leaders are loaded with egotism along with manipulative intentions; individuals when confronted with such leaders try to avoid, make distance, engage less in interaction, and are less likely to come up with new and innovative type of work-related ideas. Among employees, exploitative leaders’ reputations plummet due to their negative evaluation for such leaders who engaged with followers and are more concerned with overloading their task (Schmid et al., 2018, 2019b). Heightened distance between leaders and followers, more absenteeism, low commitment, and low hedonism at workplace are likely to follow when employees face such leadership. Since exploitative leaders are found to perpetrate by delegating additional tasks with mounted pressure in workplace settings (Schmid et al., 2018), employees feel disrespect, dehumanized, and are likely to indulge in facade creation to get relief from, under such a situation, extra tasks. A more self-lover and egoistic exploitative leader tend to gratify himself/herself with attainment of his/her personal goal achievement on the cost of followers hard work; consequently employees might perceive that their inner self being ruined thus are not be able to produce creative value-driven ideas. Since exploitative leaders are not habitual to offer liberty to followers, they hamper their cognitive development by assigning average or even below-the-line tasks (Schmid et al., 2019b), employees labeled these leaders as opportunistic who undermines their competency level, which encourages them to create the FOC. According to the study by Hobfoll et al. (2018), when people experienced actual loss or threatened of resource loss, then they experience strain. Guo et al. (2020) suggested that EL consumes followers’ resources such as social support, self-esteem, and job control. Indeed, when employees witnessed exploitation from leaders, then they feel a threat to lose their psychological resources (Schmid et al., 2019a). Indeed, it is suggested that EL facilitates the adoption of FOC. Previous studies reported that EL increases psychological distress (Majeed and Fatima, 2020), knowledge hiding, and turnover intentions (Syed et al., 2021). Therefore, it is suggested that followers when confronted with exploitative leader’s behavior tend to embrace FOC.

According to COR, individuals are highly sensitive about the loss of valued resources (Halbesleben et al., 2014). Resource loss is more salient than resource gain, and for this very reason, individuals try to protect their resources, i.e., personal skills and personal traits (Hobfoll et al., 2018). As per COR, when followers face acute losses in resource, then they experienced anxiety and want to protect their remaining resources (Hobfoll, 2001). Guo et al. (2020) stated that EL consumes individual resources. Thus, when leaders exercise exploitative style, then followers experience FOC and absenteeism as a coping strategy, i.e., to protect their resource (Magee et al., 2017). Because when they perceive the threat of resource loss from the supervisory side (i.e., exploitative leader), they are engaged in the facade of conformity to protect their valuable resources. According to COR theory (Hobfoll, 1989), people want to attain, sustain, and reserve resources. COR theory posits that resource loss is more powerful than resource gain in magnitude and tends to affect people more rapidly and at an increasing speed over time (Hobfoll et al., 2018). So, to protect their valuable resources, individuals indulge in increasing absenteeism (i.e., lateness at workstation) because they feel threatened to lose their resources (Ahmad and Begum, 2020) under an exploitative leader. Thus, when followers experience manipulation under an exploitative leader, they tend to protect their valuable resources by engaging in conformity facades, which then leads to employee absenteeism as a coping mechanism. Thus, it is hypothesized that:

H2: *The relationship between exploitative leadership and absenteeism is mediated by facades of conformity.*

### Mediating Role Depression

It is also vital to understand how effective coping strategies can alleviate the exploitation impact (Schmid et al., 2018, 2019b). EL termed to be the main reason for social support loss. Likewise, followers who are victimized to consistent exploitation termed such loss as a loss of autonomy and loss of job control. As exploitative leaders consistently assign boring and unmatched tasks to followers, elusive work pressure evolves (Schmid et al., 2018). Thus, such individuals are likely to make the employee depressive and sadist. Drawing on this theoretical reasoning, this research predicts leaders’ exploitation being a stressor which instigates a negative impact on followers’ resources and thus tandem psychological stress and tension to meet job demands. A significant form of psychological tension studied in previous research is depression (Harvey et al., 2007).

Exploitation significantly affects mood causing several reasons. First, exploitation at the workplace produces a painful and negative experience, while studies examined that intense emotional reactions such as pain and tension might be prompted due to negative experiences (Taylor, 1991). In addition, such oppression might increase if employees are confronting a stressor at the workplace (Choi, 2019). Evident in the findings of earlier studies is that apostates are miserable and depressed (O’leary, 1990; Ferris et al., 2008). Previous studies reported that workplace stressors might threaten employee’s psychological wellbeing and increase their risk for mental health problems, such as depression (Luo et al., 2016; Han et al., 2017).

Overall, EL has been found to be a fatal workplace stressor that posits threats to employees’ resources, their wellbeing, and the ability to perform tasks (Syed et al., 2019b). Under depressive work environment, employees’ resources reduce rapidly, and resource replenishing might not be useful for all due to different personalities (Zhou et al., 2018). Specifically, exploited employees experience more negative self-evaluation about themselves, such as discouragement and inferiority. These negative self-evaluation leads to depression. In the same line of reasoning, previous research revealed that depression has a positive and significant effect on various withdrawal behaviors (Pollack et al., 2012). Experiencing stressor at the workplace (i.e., EL) can cause poor mental health (i.e., depression) (Sawhney et al., 2018; Bartoll et al., 2019), which is likely to result in increased absenteeism in employees from work. Hence, based on the aforementioned arguments, it is hypothesized that:

H3: *Depression mediates the relationship between exploitative leadership and employee absenteeism.*

### Serial Mediation

**FIGURE 1:**
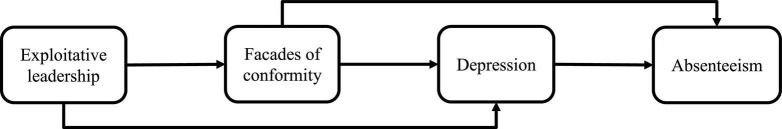
Research model.

As shown in Figure 1, and in line with studies that discussed EL (Schmid et al., 2018, 2019b) and employee absenteeism (Nevicka et al., 2018), this study aimed to establish these relationships in a serial mediation model where it is suggested that how EL is linked to employee absenteeism *via* FOC and depression. Previous studies reported that a negative form of leadership might directly increase employee absenteeism due to his/her social relationship with subordinates. Still, this above relationship might be influenced indirectly as well (Nevicka et al., 2018). Individuals exposed to exploitative leaders tend to reduce further loss of other resources, therefore are prone toward disengagement with task and withdraw themselves as a coping strategy (Shirom, 2003). Therefore, depletion of resources, combined with withdrawal from one’s tasks, is likely to result in high absenteeism. Specifically, it is argued that under EL, subordinates are entitled to engage in creating facades (i.e., mask themselves or hide their true self) to protect their valuable resources from exploitation. Also, when such exploited individuals indulged in facade creation perceive insecurity about their new ideas/inputs, it is likely to result in poor mental health (e.g., depression), which ultimately leads to absenteeism. Therefore, we predicted serial mediation hypothesis as:

H4: *Facades of conformity and depression sequentially mediate the relationship between exploitative leadership and absenteeism.*

## Research Method

### Study Design and Participants

This study developed and tested the serial mediation model. The data were collected from telecom sector employees with the help of a self-administered paper-based survey questionnaire. This study follows the time-lagged (i.e., three waves) and single-source (self-report) design. Previous studies reported that self-reported data might lead to CMB (Podsakoff et al., 2003). To minimize CMB, we applied multiple methodological and statistical analyses recommended by Conway and Lance (2010), (a) providing the justification why self-reports are appropriate, (b) using proactive measure, and (c) giving the construct validity of the measure.

First, this study will explore the consequences of EL in the education sector. Self-report measures are appropriate for the participants’ assessments of their immediate supervisor who displays exploitative behavior (e.g., manipulative, egoistic, taking credit, and undermining them). EL, FOC, depression, and absenteeism are commonly perceived as subjective. For this, obtaining self-report response seems adequate. Second, we divided the survey into three different time lags with a four-week interval in each. In Time 1, we distributed 440 surveys comprising the respondents’ demographic information (e.g., survey unique ID or name, gender, job details, organization, marital status, and education), EL (i.e., self-reported) items, and received back 380 surveys wholly filled. After a four-week gap of receiving the first survey, in Time 2, we distributed the 380 surveys FOC (i.e., self-reported) among the same respondents (identified with the help of survey unique ID or name) and found 335 filled surveys. Furthermore, after the four-week interval in Time 3, we distributed the 335 surveys asking questions on depression and employee absenteeism (i.e., self-reported) from same participants. In sum, a total of 211 complete questionnaires were obtained comprising a response percentage of 48%. Finally, to justify the construct validity, we performed the conformity factor analysis for the structural model, and the results show that fit indices [x^2^ (192) = 570.11, comparative fit index (CFI) = 0.95, Tucker–Lewis index (TLI) = 0.94, root mean square error of approximation (RMSEA) = 0.08] are better than those of other models [x^2^ (210) = 3512.40, CFI = 0.50, TLI = 0.45, RMSEA = 0.27] (refer to Table 1).

**TABLE 1:** Measurement model comparison.

Measurement models		χ *^2^*	*Df*	χ ^2^/Df	*TLI*	*CFI*	*GFI*	*RMSEA*
1	EL and FOC (2 factor) EL and FOC (1 factor)	53.83	8	6.72	0.90	0.94	0.92	0.16
		1086.41	9	120.71	0.20	0.52	0.51	0.68
2	FOC and Depression (2 factor) FOC and Depression (1 factor)	211.77	8	26.47	0.43	0.70	0.81	0.34
		1083	14	77.40	0.33	0.56	0.53	0.55
3	Depression and Absenteeism (2 factor) Depression and Absenteeism (1 factor)	63.71	13	4.90	0.92	0.89	0.93	0.13
		1343.98	54	24.88	0.49	0.59	0.48	0.31
4	Full Model (4 factor) Full Model (1 factor)	**570.11**	**192**	**2.96**	**0.95**	**0.96**	**0.84**	**0.08**
		3512.40	210	16.72	0.45	0.50	0.41	0.27

*Better fit indices are presented in bold; full model (4-factor model) combines exploitative leadership, facades of conformity, depression, and absenteeism.*

### Variable Measurement

To collect the data, this study adopted the existing valid measures of the study constructs. The questionnaire was administered in English, as language was not an issue and is the official business language in Pakistan (Akhtar et al., 2020a,b; Javed et al., 2021). All the measures are anchored on a 5-point Likert scale. Measures for study constructs were adopted from previous studies in line with the operational definitions of the variables.

Exploitative leadership: We used 15-item scale of Schmid et al. (2019a) to measure exploitative leadership at Time 1. The Cronbach alpha reliability of this instrument is found to be 0.78. The sample items include: *“Takes it for granted that my work can be used for his or her personal benefit”* and *“Puts me under pressure to reach his or her goals.”*

Facades of conformity: In this study, we have used a 6-item scale to measure FOC originally developed by Hewlin (2009). The alpha reliability of scale is found to be 0.83, whereby items are, “*I don’t share certain things about myself to fit in at work”* and *“I suppress personal values that are different from those of the organization*.”

Depression: A 9-item Patient Health Questionnaire (PHQ) developed by Kroenke et al. (2001) was adopted to tap depression at Time 3; the reliability of this instrument is found to be 0.80. A sample question includes “*during the last month, how often were you bothered by feeling down, depressed, or hopeless?”* Answers were measured on a response scale, 1 (i.e., not at all), 2 (i.e., several days), 3 (i.e., every week), 4 (i.e., more than half the days), and 5 (i.e., nearly every day).

Absenteeism: We measured employee absenteeism at Time 3 by using the 5-item scale, three items from Geurts et al. (1994) and two items from Autry and Daugherty (2003). The sample question is “*I have been absent for reasons associated with work stress this year*” and “*How often have you been absent from the job because you just didn’t feel like going to work?*” The reliability of the scale is 0.78. All the items were measured on a 5-point Likert scale ranging from 1 “never” to 5 “very frequent; every day.”

## Results

Table 2 shows estimates for all study variables, i.e., descriptives, correlations, and reliability statistics, along with the value of means and standard deviations. The Cronbach alpha’s result also reported that all the variables’ measurement scales are reliable, having a value of above 0.70, and the cutoff value is recommended by Nunnally (1982).

**TABLE 2:** Correlation analysis results.

Sr. no		Mean	SD	1	2	3	4
1	Exploitative leadership	3.26	1.29	***(0.94)***			
2	Facades of conformity	3.15	0.47	0.39**	***(0.70)***		
3	Depression	2.81	0.64	0.65**	0.40**	***(0.70)***	
4	Employee Absenteeism	2.96	0.50	0.69**	0.65**	0.70**	***(0.87)***

N = 211; bold values represents alpha reliabilities are presented in parentheses, **p < 0.01.

All the variables of the study were conducted at different time lags. EL has a significant positive correlation with FOC (*r* = 0.40, *p* < 0.01), depression (*r* = 0.65, *p* < 0.01), and employee absenteeism (*r* = 0.69, *p* < 0.01). Also, FOC has a significant and positive correlation with depression (*r* = 0.40, *p* < 0.01) and employee absenteeism (*r* = 0.65, *p* < 0.01). Finally, depression has a significant and positive correlation with employee absenteeism (*r* = 0.70, *p* < 0.01).

For testing direct, indirect, and serial mediation hypotheses (i.e., H1, H2, H3, and H4), this study employed PROCESS Macro by Hayes (2015). We performed Model 4 for simple mediation and Model 6 for serial mediation, respectively, in PROCESS Macro (refer to Table 3). The mediation technique used by Hayes’ “*directly tests the indirect effect between the predictor and the criterion variables through the mediator via a bootstrapping procedure, addressing some weaknesses associated with the Sobel test*” (Van Jaarsveld et al., 2010). Furthermore, it provides bootstrapped confidence intervals (CIs) and associated statistical significance tests for indirect paths (Warner, 2013). Therefore, in this study, we validated the indirect effects (ELs) and its effect on ABS *via* FOC and depression.

**TABLE 3:** Mediation analysis.

		*M*^1^ (FOC)				*M*^2^ (Depression)				*Y* (Absenteeism)		
		Coeff.	SE	*P*		Coeff.	SE	*p*		Coeff.	SE	*p*
Exploitative Leader (EL)	a^1^	0.14	0.02	0.000	a^2^	0.29	0.03	0.001	c^,1^	0.12	0.02	0.001
Facades of conformity (FOC)		–	–	–		0.23	0.08	0.01	b^1^	0.41	0.05	0.001
Depression		–	–	–		–	–	–	b^2^	0.27	0.04	0.001
Constant	i^1^	2.68	0.08	0.000	i^2^	1.14	0.22	0.000		0.52	0.14	0.001
		R^2^ = 0.16				R^2^ = 0.45				R^2^ = 0.70		
Indirect effects (EL on Absenteeism)						Effect [95% CI]						
Indirect effect *via* FOC										0.07* [0.04 0.10]		
Indirect effect *via* Depression										0.11** [0.07 0.15]		
Serial mediation (EL→FOC→ Depression → Absenteeism)									0.008* [0.006 0.018],			

N = 211 for self-reported outcome. *p ≤ 0.05. **p ≤ 0.01 s. Bold values are confidence intervals.

As evident from results, EL has a positive relationship with absenteeism (*b* = 0.20, *t* = 3.75, *p* < 0.01) (Table 3). Thus, H1 is supported. H2 stated that EL and absenteeism relationship is mediated by FOC. According to Table 3, EL has an indirect effect on absenteeism *via* FOC (*b* = 0.07*, SE = 0.03, Z = 1.98, *p* < 0.05) as the indirect effect of EL on employee absenteeism *via* FOC did not include zero [β = 0.07, CI (04, 0.10)], which provides support to H2. As indicated in Table 3, EL has an indirect effect on absenteeism *via* depression (*b* = 0.11*, SE = 0.03, *z*-value = 2.05, *p* < 0.05) as the indirect effect of EL on employee absenteeism *via* depression did not include zero [β = 0.11, CI (07, 0.115)], which provide support to H3. Also, the result indicates that EL has an indirect effect on absenteeism *via* FOC and depression (*b* = 0.08^**^, SE = 0.01), which was found to be substantiated at 95% CI (0.006, 0.018). Therefore, hypothesis 4 was substantiated.

## Discussion

In the present highly competitive workplace environment, EL has been considered as an acute threat and risk for employees (Schmid et al., 2018, 2019b; Syed et al., 2019a), although it is still in infancy that how and why it happens in organizations (Schmid et al., 2018, 2019b). By addressing this important question, we investigated the effects of EL on employee absenteeism; furthermore, the impact of EL on employee absenteeism is serially mediated by FOC and depression.

This study filled an important gap in the literature, as limited studies investigated the outcomes of EL (Schmid et al., 2018, 2019b; Syed et al., 2019a). Therefore, this study examined the effects of EL on employees’ absenteeism. This study’s findings revealed that leaders’ exploitative behavior pushes the followers toward absenteeism because exploitative leaders manipulate followers and do not care about followers’ development. The findings of the this study aligned with previous studies, which reported that EL positively affects employee behaviors (Schmid et al., 2018, 2019b). Theoretically, when employees face workplace stressors (i.e., EL in our case), they tend to cope with the situations to prevent their resources (i.e., employee absenteeism as a coping strategy). This is in accordance with the theoretical assumptions of COR theory (Hobfoll, 1989, 2001, 2011; Halbesleben et al., 2014).

This study’s findings also reveal that FOC mediates the relationship between EL and employee absenteeism. In circumstances where employees observe that their leader takes all the credit for their hard work, manipulates them, and does not show much care about the development of their followers, then followers may hesitate to share new ideas, which means they masked their true self and portray behavior according to leader demand/requirement. This will lead to employee absenteeism at the workplace. According to the COR theory, individuals try to protect their resources from further loss when they have or perceive the theft of loss (Hobfoll, 2001, 2011).

Findings also reveal that depression mediates the relationship between EL and employee absenteeism. When followers continuously encounter the exploitative leader, who is focused on his personal goal achievement based on followers’ efforts and not cares about the growth of followers, then the followers may experience depressive systems, which will ultimately dampen their interest at job and organization, so they are more inclined toward absenteeism. As per the assumptions of COR, under stressful conditions (i.e., exploitative leaders), followers may engage in the protection of their resources so they may experience cognitive disorder (i.e., depression), which will lead them to absenteeism at the workplace.

Overall, we found good support for all our proposed hypotheses. Interestingly, this study’s insights are in line with the earlier studies in domain and verdicts that destructive leadership behaviors bring negative consequences for individuals (Neves and Schyns, 2018). Notably, such findings validate previous studies suggesting that leadership influences follower outcomes through different underlying processes (Zhang and Bartol, 2010; Zhang et al., 2012).

### Theoretical Implications

This study embeds several contributions to theory. First, this study contributes to nascent domain in the EL literature by identifying its new outcome, i.e., absenteeism. Recently, studies paid their attention to determine the outcomes of EL (Schmid et al., 2018, 2019b; Guo et al., 2020; Majeed and Fatima, 2020). Perhaps, Schmid et al. (2018) stated that EL positively related to turnover intentions. Schmid et al. (2019b) apprised that EL brings gradual loss in followers’ satisfaction with job and affective commitment. Majeed and Fatima (2020) stated that EL increased psychological stress *via* negative affectivity. Syed et al. (2021) concluded that EL decreased employee performance and creativity *via* knowledge hiding. In fact, this emerging area predicting EL on absenteeism has often fallen in backburner and overlooked in organizational behavior studies. Therefore, this study broadens the EL literature by substantially relating it with absenteeism.

Second, the mediating role of FOC and depression in such underlying mechanism further advances and broadens the literature regarding EL with its subsequent outcomes. Earlier studies found a substantiated relationship for direct effects of EL (Schmid et al., 2018, 2019b), but relatively failed to scrutinize the EL mechanism in such depth and detail (Syed et al., 2021). Based on the COR theory contentions, this study indicates that FOC and depression are important mediating mechanisms between EL and absenteeism. Findings reveal that EL, a distinctive resource-draining leadership style, increases followers’ FOC and depression and subsequently leads to absenteeism. Akhtar et al. (2020b) found similar results and further corroborated that FOC mediates the influence of supervisor ostracism and unethical work behavior. In addition, this study considered theoretical assumptions of COR as useful to comprehend EL and its effects, thereby highlighting such psychological processes by which EL affects negative outcomes.

### Managerial Implication

This study offers several practical implications. First, the results reveal that under EL, subordinates/followers are more likely to engage in FOC (just to suppress their feeling), which will lead to employee absenteeism. However, previous studies reported that followers engage in surface acting under dark leaders just to avoid the conflict (Tepper, 2007). That is why, we suggested that, to deal with the absenteeism issue, employees need cognitive effort in dealing with difficult situations such as EL and attend organizations-arranged training programs on emotional regulation skills in the leader-follower relationship.

Employees should be keen and be able to learn emotional regulation strategies to engage with exploitative leaders; by doing so, they might be less susceptible to experience resource-depleted symptoms, i.e., FOC (Rupp and Spencer, 2006). Keeping the study results in view, managers must ensure the positive leadership style and build high-quality leader-member exchange (LMX) to avoid the FOC because a high-quality LMX relationship stimulates employees to express their true selves (Bowen, 1982). Third, human resource development (HRD) department should arrange training programs for managers to enhance their supportive and effective management style by which subordinates freely discuss and share their ideas with the supervisor without any threat.

### Limitations and Future Direction

This study has several limitations. First of all, as the data collected were self-reported, this might account for CMB in the study. Previous studies state their concern to mitigate the potential effect of CMB in such studies (Akhtar et al., 2020a; Javed et al., 2020), thereby it is advisable to obtain other reported data, i.e., supervisor and peer reported. Second, this study draws on the reasoning of COR theory to examine EL roles on employee absenteeism *via* FOC and depression. Where employees perceive exploitative leader as a threat (i.e., egoistic, undermine, taking credit, exerting pressure, and manipulating) to their resources and make it a scarce resource to meet job demand, then subordinates may be engaged in creating facades and depression, which will lead to employee absenteeism. When individuals are experiencing the exploitation situation, they are less likely to increase their resources and use coping strategies to compensate for the previous resource loss or depletion of further resources (Hobfoll, 2011). Hence, in future studies, it is suggested to undertake some different theoretical assumptions to underpin EL and its subsequent outcomes. Third, this research is carried out in developing context of Pakistan with data obtained from education sector employees. Based on the Hofstede’s insight (1983), uncertainty avoidance and high power distance are the main facets of country’s culture. Such high power distance accounts for severe situations between supervisor and subordinate to exploit as leader/supervisor usually tends to dictate what need to do. High uncertainty avoidance creates situations where people avoid taking risks. Therefore, when subordinates experience an exploitative leader who is egoistic, undermine, take credit, exert pressure, and manipulate, the followers usually adopt silence (i.e., facades) against leader misuse or exploitation. Therefore, the future studies may be conducted in other cultural contexts to unveil the effect of EL.

### Conclusion

This study concludes that EL is found to be a main reason to propagate absenteeism. Also, EL has an indirect effect on absenteeism, which passes through FOC and depression. COR theory offers a crucial role in validating these aforementioned relationships.

## Data Availability Statement

The raw data supporting the conclusions of this article will be made available by the authors, without undue reservation.

## Ethics Statement

The studies involving human participants were reviewed and approved by COMSATS University Islamabad (CUI), Sahiwal Campus constitutes Campus Ethics Approval Committee. Written informed consent for participation was not required for this study in accordance with the national legislation and the institutional requirements.

## Author Contributions

MWA suggested the idea of this research and wrote the initial protocol of this study. CH developed the conceptual framework. FS and MAS performed the statistical analysis of data. AR, MH, MA, and MSS collected the data of the study, interpreted the data, and wrote the manuscript. All authors reviewed the manuscript and approved its final version.

## Conflict of Interest

The authors declare that the research was conducted in the absence of any commercial or financial relationships that could be construed as a potential conflict of interest. The reviewer SA declared a shared affiliation with the authors MWA and MSS to the handling editor at the time of review.

## Publisher’s Note

All claims expressed in this article are solely those of the authors and do not necessarily represent those of their affiliated organizations, or those of the publisher, the editors and the reviewers. Any product that may be evaluated in this article, or claim that may be made by its manufacturer, is not guaranteed or endorsed by the publisher.
